# Prevalence and management of laryngotracheal trauma in India

**DOI:** 10.6026/973206300222731

**Published:** 2026-05-31

**Authors:** Priyanka Verma, Aditya Gargava, Megha Prabhakar, Shiv Kumar Raghuwanshi

**Affiliations:** 1 Department of ENT, Atal Bihari Vajpayee Government Medical College, Vidisha, Madhya Pradesh, India; 2 Department of ENT, Shrimant Rajmata Vijayaraje Scindia Medical College and Hospital, Shivpuri, Madhya Pradesh, India

**Keywords:** Laryngotracheal trauma (LTT), tracheostomy, dysphonic severity index, contrast-enhanced computed tomography (CECT), road traffic accidents

## Abstract

Laryngotracheal trauma is a rare but life-threatening condition requiring prompt diagnosis and management to prevent airway compromise and long-term functional deficits. Therefore, it is of interest to evaluate the etiology, clinical presentation, management and outcomes of laryngotracheal injuries in 15 patients aged 15-60 years treated between 2021 and 2023 at a tertiary care center. Injury severity was graded using the Schaefer system and all patients underwent fibreoptic endoscopy and contrast-enhanced CT, with functional outcomes assessed using the Dysphonia Severity Index and New Zealand Secretion Scale. Most patients were male, with cut-throat injury and road traffic accidents as common causes and nearly half required emergency tracheostomy with successful decannulation. Early assessment and management resulted in favorable airway, phonatory and swallowing outcomes, highlighting the importance of fibreoptic endoscopy in guiding clinical decisions.

## Background:

The larynx is in a very vulnerable anatomical location at the anterior cervical compartment, being partially guarded by the mandible at the top and the sternum at the bottom, but is vulnerable to a broad array of destructive forces [1]. It is the centre of three vital physiological processes and functions such as respiration, phonation and deglutition and any traumatic loss in its functional integrity has far reaching consequences to life and survival [2]. Laryngotracheal trauma is a heterogenic range of injuries that can occur due to blunt force trauma, penetrating trauma, inhalational burns, corrosive ingestion, iatro-genic manipulation and sequelae of post-intubation [3]. Epidemiological predictions determine the prevalence of laryngeal trauma at about 1 in 4,000 to 1 in 40,000 emergency department presentation; the figure has generally been agreed to reflect an underestimation in view of the high prehospital fatality rates with severe injuries [4]. It has been documented that mortality rates are up to 40 per cent in blunt traumas and 7-20 per cent in penetrating ones, highlighting the potentially fatal nature of such lesions [5]. In Western populations, the majority of cases are identified by blunt mechanisms, including motor vehicle collisions and direct cervical impact, but penetrating injuries (including cut- throat wounds of suicidal, homicidal, or accidental causes) are a large percentage of cases in low- and middle-income countries [6]. The clinical manifestation of laryngotracheal trauma is infamously inconsistent and can vary on the scale of mild cervical tenderness and undetectable dysphonia to the airway blockage and hemodynamic crunch [7]. Cervical spine injury, esophagus injury and major neurovascular injury are usual and can greatly affect management choice and prognosis [8].

The most commonly used system of grading severity of laryngeal injury and guiding treatment approach is the Schaefer classification system that categorizes injuries between Grade I (minor endolaryngeal hematoma) and Grade V (laryngotracheal separation) [9]. Another factor that has significantly enhanced the accuracy and speed at which injury is assessed is the development of diagnostic imaging, specifically contrast-enhanced computed tomography and the proliferation of fibreoptic endoscopy [10]. However, some of the controversies are related to the best time of surgical intervention, indicators of stenting and the choice of fixation material to be used to remedy displaced laryngeal fractures [11]. Also, a large part of the available literature is based on high-volume trauma centres in developed countries and the information concerning the resource-starved settings in South Asia is still scarce [12]. There is a research gap in a critical evaluation of the long-term functional outcomes, including voice, swallowing and airway adequacy, after laryngotracheal trauma in the secondary and tertiary care institutions with limited infrastructure available in the form of super specialization [13]. Therefore, it is of interest to determine and study the etiological factors, demographic information and trends of laryngotracheal trauma presentation in our institution over a three-year period; and secondly, to determine the outcome of patients in terms of airway restitution, voice quality and swallowing capacities after severity-based management.

## Materials and Methods:

## Study design and setting:

The study was a retrospective observational study, to be carried out at the Department of Otorhinolaryngology, Atal Bihari Vajpayee Government Medical College (ABVGMC), Vidisha, Madhya Pradesh, India, between January 2021 and December 2023. The institution is a tertiary care referral center that serves mostly rural and semi-urban population. The Institutional Ethics Committee gave an ethical clearance before the commencement of the study. The informed written consent has been obtained in the native language of all the participants.

## Study population:

The study period had 15 consecutive patients with laryngotracheal trauma. The inclusion criteria would include patients aged 15-60 years of either sex who reported blunt laryngotracheal trauma, penetrating or cut-throat injury, corrosive ingestion injury, or injury to the laryngotracheal complex by intubation. The subjects that were not included in the research were those with isolated intrathoracic tracheal injuries.

## Clinical assessment scale:

All the patients were first seen at the emergency department and later referred to the ENT department to have a definite assessment and management. A pro forma of data was used to record demographic data, mechanism and site of injury, presenting symptoms, physical examination data, diagnostic investigations, grade of injury, airway management plan, surgery procedure done and functional result.

## Diagnostic assessment:

Diagnostic tests consisted of flexible fiberoptic nasolaryngoscopy and/or video-laryngoscopy to determine the endolaryngeal mucosa, vocal cord mobility and hematoma, edema, or mucosal disruption. All the patients who were hemodynamically stable underwent contrast-enhanced computed tomography (CECT) of the neck to assess cartilaginous fractures, soft tissue disruption and the condition of the adjacent structures. Additional imaging used were anteroposterior and lateral soft tissue neck radiographs.

## Severity grading:

Each injury was rated by the Schaefer system. Grade I refers to small endolaryngeal hematoma being not fractured. Grade II has edema, hematoma or nondisplaced fracture with no exposed cartilage. Grade III includes a massive edema, mucosal disruption, displaced fracture, immobility of the vocal cord, or uncovered cartilage. Grade IV is characterized by severe anterior laryngeal disruption and unstable fracture and widespread mucosal injury. Grade V is complete laryngotracheal division.

## Outcome assessment:

Follow-ups of patients were done starting with the admission and up to at least one year after injury. Phonatory results were measured based on the Dysphonic Severity Index (DSI) which is a multiparametric objective scale, comprising of maximum phonation time, highest fundamental frequency, lowest intensity and jitter percentage. Swallowing was also assessed using Fiberoptic Endoscopic Evaluation of Swallowing (FEES) and rated using the New Zealand Secretion Scale, a 7-point scale that measures secretion location, amount and the response of the patient.

## Statistical analysis:

All the data were transferred to the computer and processed with the SPSS version 26.0 (IBM Corporation, Armonk, NY). Means of continuous variables were used and frequencies and percentages used to represent means of categorical variables. The Chi-square test or Fisher exact test depending on the situation was used to conduct group comparisons of the categorical variables. The p-value of below 0.05 was deemed to be statistically significant.

## Results:

A total of 15 patients with laryngotracheal trauma were included in the study. The cohort comprised 11 males (73.3%) and 4 females (26.7%), with a male-to-female ratio of 2.75:1. The mean age of the patients was 35.8 ± 20.1 years. The etiological distribution of injuries is presented in Table 1. Penetrating injuries (cut-throat) were the most common cause, accounting for 6 cases (40.0%), followed by road traffic accidents in 4 cases (26.6%) and assault by strangulation in 3 cases (20.0%). Less common causes included bee sting-induced anaphylaxis and corrosive ingestion, each contributing 1 case (6.7%). Based on the mechanism of injury, blunt trauma was observed in 8 patients (53.3%), while penetrating trauma was noted in 7 patients (46.7%). Comparison of injury severity between blunt and penetrating trauma revealed no statistically significant difference (χ^2^ = 3.82, p = 0.43), although higher-grade injuries (Grades III and IV) were more frequently associated with penetrating trauma. The distribution of injury severity according to the Schaefer classification and corresponding management strategies is summarized in Table 2. Grade II injuries were the most prevalent, observed in 7 patients (46.6%), followed by Grade III in 3 patients (20.0%), Grade IV and Grade V in 2 patients each (13.3%), and Grade I in 1 patient (6.7%). Regarding management, 7 patients (46.6%) required emergency tracheostomy, while 8 patients (53.3%) were managed conservatively without surgical airway intervention. Patients with Grades III and IV injuries underwent panendoscopy and open reduction, along with tracheostomy and stenting, whereas Grade V injuries required airway stabilization and referral to a higher center. Functional outcomes following treatment are detailed in Table 3. No mortality was recorded in the study population. Among the 7 patients who underwent tracheostomy, all were successfully decannulated, yielding a 100% decannulation rate, with a mean decannulation time of 90 ± 21.5 days (range: 60-120 days). No patient required permanent tracheostomy. At final follow-up, 12 patients (80.0%) demonstrated normal voice quality, while 3 patients (20.0%) had residual dysphonia (mild in 2 patients and moderate in 1 patient). The mean Dysphonic Severity Index score was 3.2 ± 1.8, indicating predominantly normal to mildly impaired phonatory outcomes. Transient unilateral vocal cord palsy was observed in 3 patients (20.0%), all of whom recovered spontaneously during follow-up, and none required medialization thyroplasty. Swallowing outcomes were favorable, with all 15 patients (100%) achieving normal oral feeding within 6 months. Initial dysphagia was noted in 6 patients (40.0%), which resolved completely within 3 months. No patient required permanent enteral feeding. The mean New Zealand Secretion Scale score at final follow-up was 0.4 ± 0.7, indicating effective secretion control and absence of clinically significant dysphagia. The most common presenting symptom was dysphonia (53.3%), followed by dysphagia (40.0%) and stridor (6.7%). Injuries were predominantly localized between the hyoid bone and the upper trachea. No associated major craniospinal or thoracoabdominal injuries were observed in this cohort.

## Discussion:

Although laryngotracheal trauma is rather unlikely to occur in the daily otolaryngology practice, it presents a very challenging diagnostic and therapeutic challenge that requires a methodical and integrated attitude. The data of the current research will be of useful information about the clinical continuum and outcomes of management of these injuries in a tertiary care unit with limited resources. The observed male dominance in our cohort (73.3%) is quite similar to the results of the entire world literature and can be explained by the fact that men are more exposed to occupational factors, use motor vehicles more often and are among the participants of interpersonal violence in disproportions [14]. The 35.8 mean age of the current study is similar to those that have been previously reported with large series carried out in North American and European centers reporting the mean ages of 29 to 37.3 years [15]. This demographic trend highlights the socioeconomic influence of the laryngotracheal trauma by pointing out that laryngotracheal trauma affects the economically productive portion of the population. An interesting observation during this study is that the most common etiology is by cut-throat (40%), unlike in Western sources where the vast majority of cases are attributed to blunt mechanisms (mostly motor vehicle collisions) [16]. The somewhat high percentage of penetrating trauma in our sample probably represents aspects of local socio-demographics, such as the high incidence of suicidal neck trauma and homicidal neck trauma in Central India. This etiological trend has been reported in other similar studies in South Asian institutions where penetrating neck injury especially self-inflicted cut-throat injuries make up a large percentage of laryngeal trauma admissions [17]. The second mechanism (26.6 percent) was the road traffic accidents, which was in line with their proven significance as major cause of blunt laryngotracheal injury across the globe [18]. Creation of a safe airway is the most important intervention to the management of laryngotracheal trauma. The airway management choice should balance between the urgency of airway compromise and the risk of iatrogenic harm of the blind or forceful instrumentation [19]. Although endotracheal intubation is suitable in case of minor injuries to the supraglottic, it is associated with high chance of transformation of partial airway blockage to complete in case of severe end laryngeal disruption. Tracheostomy via local anesthesia is the safest mode of practice in such situations [9]. Emergency tracheostomy was conducted in 46.6 percent of the patients in our series and the remaining patients were taken care of by means of conservative observation and monitoring. The 100 percent decannulation rate of our study is similar to results published and is consistent with the overall positive outcome of lower-grade injuries when treated with timely airway intervention [20]. The Schaefer typology gave a practical and functional guideline to guide the management in our group. Grade I and II (53.3% of the cases) were managed by conservative therapy using corticosteroids, anti-reflux therapy, humidification and close endoscopic follow-up, which was supported by evidence of excellent airway and phonatory results in non-displaced non-operative-managed fractures [21]. Grade III and IV injuries also involved open surgical debridement with fracture correction and mucosal sutures and used with stenting where there was an offset or unstable cricoid fracture. The controversy concerning the best fixation technique-suture and miniplate - is yet to be resolved, but with current evidence showing that rigid fixation using miniplates stimulates cartilaginous union and possibly better long run structure results than suture fixation, which forms fibrous healing [22]. When surgical exploration is best after laryngotracheal trauma has been an issue of long-standing controversy. Advocates of early intervention believe that exploration of 24-48 hours will avoid the development of cicatricial tissue and granulation, which hinder the accurate reduction of anatomy [23]. Large case series studies have revealed that surgical procedures conducted within the 48 hours of injury have good results in around 80 percent of cases and significantly poor results in cases where surgery is undertaken after 48 hours [24]. We had an institutional culture of using early exploration as a method of treating Grade III and IV injuries when hemodynamic stability allowed and the positive functional outcomes of this practice confirm the use of it. Our study has promising phonatory and swallowing results. Eighty percent of patients had normal voice quality at the end of the follow-up and three patients with temporary unilateral vocal cord palsy had a complete spontaneous recovery. This pattern of recovery is in line with the natural history of recurrent laryngeal nerve neuropraxia after blunt cervical trauma where most of them are resolved within six months [25]. The complete lack of medialization thyroplasty in our cohort is another witness to rather positive nerve recovery perspectives when the injury mechanism is not associated with transeunt injury. On oral feeding, all the patients achieved normal feeding within six months and from the six patients with dysphagia at the beginning, all patients showed total improvement in three months. The consumption of rehabilitation after laryngotracheal trauma has not yet been addressed in the literature as much as phonatory rehabilitation, although it is also very important to the quality of life of patients [26]. Fibreoptic endoscopic swallowing assessment using a fibreoptic deglutition secretion scale as an objective and repeatable measure of deglutition was applied to our cohort. There are a few weaknesses that should be recognized. The retrospective design and the limited sample size do not allow conclusive results to be made on the effectiveness of one management style over another. The study is single- centered, which reduces the extrapolation ability of the results. Moreover, the lack of a referral in the two most complicated injuries (Grade V), which demanded a referral, was also a limitation at our institution due to the lack of services such as the absence of super specialty services, miniplate fixation systems, or silicone stents. Evidence-based management protocols to be used in different clinical settings should be determined by larger, multicentric prospective studies using standardized outcome measures and extended follow-up periods [27].

## Conclusion:

Laryngotracheal trauma is a serious condition that requires early diagnosis, accurate severity grading and timely intervention to achieve optimal outcomes. The Schaefer classification system is effective for guiding management and fiberoptic endoscopy plays a crucial role in diagnosis and treatment. A multidisciplinary approach is essential to ensure favorable airway, phonatory and swallowing outcomes.

## Figures and Tables

**Table 1:** Etiological distribution of laryngotracheal injuries (n = 15)

**Etiology**	**Number of Patients**	**Percentage (%)**
Cut-throat / Penetrating Injury	6	40
Road Traffic Accidents	4	26.6
Assault (Strangulation)	3	20
Bee Sting (Anaphylaxis with Laryngeal Edema)	1	6.7
Corrosive Ingestion	1	6.7
Total	15	100

**Table 2:** Distribution of injury severity and management approach (n = 15)

**Schaefer Grade**	**Description**	**Patients, n (%)**	**Management Approach**
I	Minor endolaryngeal hematoma, no fracture	1 (6.7)	Conservative observation, corticosteroids, voice rest
II	Edema, hematoma, nondisplaced fracture, no exposed cartilage	7 (46.6)	Conservative management, ICU monitoring ≥24 h
III	Extensive edema, mucosal disruption, displaced fracture, vocal cord immobility	3 (20.0)	Panendoscopy, open reduction, tracheostomy, stenting (4 weeks)
IV	Severe anterior laryngeal disruption, unstable fracture	2 (13.3)	Open reduction, tracheostomy, stenting (2 weeks)
V	Laryngotracheal separation	2 (13.3)	Airway secured (intubation/tracheostomy), referred to higher center

**Table 3:** Functional outcomes following laryngotracheal trauma management (n = 15)

**Outcome Parameter**	**Result**	**Percentage (%)**
Mortality	0	0
Permanent tracheostomy	0	0
Successful decannulation (of 7 tracheostomized)	7	100
Mean decannulation time (days)	90 ± 21.5	-
Normal voice at final follow-up	12	80
Dysphonia at final follow-up (mild/moderate/severe)	3 (2/1/0)	20
Transient unilateral vocal cord palsy (all recovered)	3	20
Medialization thyroplasty required	0	0
Normal oral feeding achieved (within 6 months)	15	100
Initial dysphagia with complete resolution (< 3 months)	6	40
Permanent enteral feeding	0	0
Mean DSI score at final follow-up	3.2 ± 1.8	-
Mean New Zealand Secretion Scale score at final follow-up	0.4 ± 0.7	-

## References

[1] Shakrawal N (2022). Indian J Otolaryngol Head Neck Surg..

[2] Kaintura M (2022). Braz J Otorhinolaryngol..

[3] Cheung PKF (2021). Int J Pediatr Otorhinolaryngol..

[4] Sachdeva K, Vatsyayan R (2023). Indian J Otolaryngol Head Neck Surg..

[5] Herrera MA (2020). Colomb Med (Cali)..

[6] Arens C, MÜller AH (2023). HNO..

[7] Kamidani R (2021). Trauma Case Rep..

[8] Sargunaraj JJE (2023). Indian J Otolaryngol Head Neck Surg..

[9] Debs SL (2023). Cureus..

[10] Schweiger T (2021). Eur J Cardiothorac Surg..

[11] Alharbi SM (2025). Ear Nose Throat J..

[12] Tsai SC, Lin FC (2020). Medicine (Baltimore)..

[13] Brascia D (2023). Front Surg..

[14] ElSobki A, El-Kholy N (2023). J Laryngol Otol..

[15] Malvi A, Jain S (2022). Cureus..

[16] Mehta R (2022). J Family Med Prim Care..

[17] Alnemare AK (2024). J Clin Med..

[18] Fiz I (2020). Laryngoscope..

[19] Coulter M (2023). BMJ Mil Health..

[20] Virk RS (2019). Otolaryngol Head Neck Surg..

[21] Muraleedharan M (2022). J Laryngol Otol..

[22] Fehervari M (2021). Obes Surg..

[23] Kisser U (2022). HNO..

[24] Blumenthal D (2023). Int J Pediatr Otorhinolaryngology..

[25] Schaefer SD (1991). Arch Otolaryngol Head Neck Surg..

[26] Dewan K (2020). PLoS One..

[27] Chang MT (2021). Ear Nose Throat J..

